# Supplementary material for: Modeling glioblastoma heterogeneity as a dynamic network of cell states
Source: Mol Syst Biol. 2021 Sep 16;17(9):e10105. doi: 10.15252/msb.202010105 (PMC8444284; doi:10.15252/msb.202010105)
Supplement: Supplementary file 5 — Source Data for Figure 3 [file MSB-17-e10105-s001.zip › Figure3A_sourcedata/GSEA_3065/hallmarks_state5.GseaPreranked.1623416302988/HALLMARK_MYC_TARGETS_V2.html]

Details for gene set HALLMARK\_MYC\_TARGETS\_V2[GSEA]

|  || Dataset | state5 |
| Phenotype | NoPhenotypeAvailable |
| Upregulated in class | na\_neg |
| GeneSet | HALLMARK\_MYC\_TARGETS\_V2 |
| Enrichment Score (ES) | -0.5234192 |
| Normalized Enrichment Score (NES) | -1.6179541 |
| Nominal p-value | 0.0055555557 |
| FDR q-value | 0.014272753 |
| FWER p-Value | 0.119 |
Table: GSEA Results Summary

  

Fig 1: Enrichment plot: HALLMARK\_MYC\_TARGETS\_V2      
 Profile of the Running ES Score & Positions of GeneSet Members on the Rank Ordered List

  

| PROBE | GENE SYMBOL | GENE\_TITLE | RANK IN GENE LIST | RANK METRIC SCORE | RUNNING ES | CORE ENRICHMENT || 1 | TMEM97 |  |  | 995 | 0.211 | -0.0809 | No |
| 2 | SLC19A1 |  |  | 1198 | 0.185 | -0.0768 | No |
| 3 | DDX18 |  |  | 2288 | 0.093 | -0.1853 | No |
| 4 | NOP56 |  |  | 2390 | 0.086 | -0.1842 | No |
| 5 | MRTO4 |  |  | 2395 | 0.086 | -0.1723 | No |
| 6 | SUPV3L1 |  |  | 2959 | 0.055 | -0.2275 | No |
| 7 | UTP20 |  |  | 3228 | 0.040 | -0.2517 | No |
| 8 | MYBBP1A |  |  | 3380 | 0.032 | -0.2640 | No |
| 9 | PPAN |  |  | 3704 | 0.020 | -0.2972 | No |
| 10 | NOC4L |  |  | 3786 | 0.016 | -0.3040 | No |
| 11 | TFB2M |  |  | 3890 | 0.012 | -0.3138 | No |
| 12 | NIP7 |  |  | 4401 | -0.010 | -0.3693 | No |
| 13 | MCM4 |  |  | 4534 | -0.015 | -0.3819 | No |
| 14 | LAS1L |  |  | 4555 | -0.016 | -0.3818 | No |
| 15 | MYC |  |  | 4656 | -0.020 | -0.3901 | No |
| 16 | WDR43 |  |  | 4725 | -0.023 | -0.3944 | No |
| 17 | PRMT3 |  |  | 4746 | -0.024 | -0.3932 | No |
| 18 | PPRC1 |  |  | 4879 | -0.028 | -0.4039 | No |
| 19 | GRWD1 |  |  | 4924 | -0.030 | -0.4045 | No |
| 20 | TCOF1 |  |  | 5175 | -0.039 | -0.4268 | No |
| 21 | TBRG4 |  |  | 5300 | -0.044 | -0.4343 | No |
| 22 | WDR74 |  |  | 5427 | -0.049 | -0.4413 | No |
| 23 | BYSL |  |  | 6058 | -0.077 | -0.5007 | No |
| 24 | MPHOSPH10 |  |  | 6262 | -0.088 | -0.5107 | Yes |
| 25 | RABEPK |  |  | 6296 | -0.090 | -0.5014 | Yes |
| 26 | PES1 |  |  | 6328 | -0.091 | -0.4917 | Yes |
| 27 | IMP4 |  |  | 6345 | -0.092 | -0.4802 | Yes |
| 28 | EXOSC5 |  |  | 6442 | -0.098 | -0.4768 | Yes |
| 29 | UNG |  |  | 6479 | -0.100 | -0.4664 | Yes |
| 30 | AIMP2 |  |  | 6494 | -0.101 | -0.4535 | Yes |
| 31 | MCM5 |  |  | 6553 | -0.105 | -0.4448 | Yes |
| 32 | PLK1 |  |  | 6581 | -0.107 | -0.4324 | Yes |
| 33 | NOP16 |  |  | 6691 | -0.113 | -0.4283 | Yes |
| 34 | CBX3 |  |  | 6756 | -0.118 | -0.4185 | Yes |
| 35 | NDUFAF4 |  |  | 6970 | -0.135 | -0.4229 | Yes |
| 36 | RRP9 |  |  | 7517 | -0.193 | -0.4563 | Yes |
| 37 | GNL3 |  |  | 7548 | -0.197 | -0.4312 | Yes |
| 38 | FARSA |  |  | 7603 | -0.204 | -0.4078 | Yes |
| 39 | NOLC1 |  |  | 7699 | -0.218 | -0.3870 | Yes |
| 40 | PHB |  |  | 7753 | -0.226 | -0.3604 | Yes |
| 41 | DCTPP1 |  |  | 8303 | -0.356 | -0.3706 | Yes |
| 42 | PA2G4 |  |  | 8320 | -0.360 | -0.3206 | Yes |
| 43 | HSPD1 |  |  | 8418 | -0.393 | -0.2749 | Yes |
| 44 | CDK4 |  |  | 8625 | -0.510 | -0.2245 | Yes |
| 45 | HSPE1 |  |  | 8650 | -0.524 | -0.1517 | Yes |
| 46 | NPM1 |  |  | 8706 | -0.566 | -0.0764 | Yes |
| 47 | SRM |  |  | 8880 | -0.746 | 0.0116 | Yes |
Table: GSEA details [plain text format]

  

Fig 2: HALLMARK\_MYC\_TARGETS\_V2: Random ES distribution      
 Gene set null distribution of ES for **HALLMARK\_MYC\_TARGETS\_V2**
